# Role and Mechanism of Gut Microbiota in Human Disease

**DOI:** 10.3389/fcimb.2021.625913

**Published:** 2021-03-17

**Authors:** Yinwei Chen, Jinghua Zhou, Li Wang

**Affiliations:** ^1^School of Medicine, Hangzhou Normal University, Hangzhou, China; ^2^Institute of Aging Research, School of Medicine, Hangzhou Normal University, Hangzhou, China; ^3^School of Medicine, Zhejiang University, Hangzhou, China; ^4^Department of Evolutionary Studies of Biosystems, School of Advanced Sciences, Graduate University for Advanced Studies (SOKENDAI), Hayama, Japan

**Keywords:** gut microbiota, neurodegenerative diseases, cardiovascular diseases, metabolic diseases, gastrointestinal diseases

## Abstract

The human gut microbiome is a huge microbial community that plays an irreplaceable role in human life. With the further development of research, the influence of intestinal flora on human diseases has been gradually excavated. Gut microbiota (GM) dysbiosis has adverse health effects on the human body that will lead to a variety of chronic diseases. The underlying mechanisms of GM on human diseases are incredibly complicated. This review focuses on the regulation and mechanism of GM on neurodegenerative diseases, cardiovascular diseases, metabolic diseases and gastrointestinal diseases, thus providing a potential target for the prevention and treatment of disease.

## Introduction

The human gut microbiota (GM) is a complex, dynamic, and spatially heterogeneous ecosystem inhabited by a myriad of microorganisms interacting with each other and with the human host, including bacteria, fungi, archae, and viruses. The collection of all intestinal microorganisms genes represent a genetic repertoire, which is one order of magnitude higher than that of human genome (Fan and Pedersen, 2021). To some extent, it is also considered as the “essential organ” of human body (Ding et al., 2019). As the largest micro-ecosystem in the human body, GM is symbiotic with the host and maintains normal physiological processes in a dynamic equilibrium state. The composition of GM mainly includes four categories, including Firmicutes, Bacteroides, actinomycetes, and Proteus. *Firmicutes/Bacteroidetes* ratio is an important parameter to reflect GM disorder (Turnbaugh et al., 2006; Turnbaugh et al., 2009; Thaiss et al., 2016). In addition, the abundance, diversity and evenness of GM are also important indicators to reflect the composition of intestinal flora.

Once the GM disorder occurs, the structure and function of the intestinal flora will change and even cause the occurrence or development of some diseases. The underlying regulatory mechanisms are complex. In recent years, with the rapid development of molecular biology, genomics, bioinformatics analysis technology, and high-throughput sequencing technology, the research of GM has made rapid progress. A great deal of research has produced evidence that GM disorder and its metabolites play a key role in maintaining host intestinal homeostasis and influencing the development of many diseases, including neurodegenerative diseases, cardiovascular diseases, metabolic diseases, and gastrointestinal diseases.

The imbalance of GM will affect the health of the host through many ways, such as energy absorption, choline, short chain fatty acids (SCFAs), gut-brain axis, bile acids (BAs), and so on. However, the mechanism of GM on human diseases has not yet been completely elucidated. In this paper, we review the latest findings related to the regulation and mechanism of GM on human diseases, then summarized the characteristics of intestinal microflora changes associated with various diseases (**Supplementary Table**), and the possible mechanisms of microbial metabolism and derivatives involved in driving the occurrence and development of diseases. Thus providing a potential target for prevention and treatment of disease.

## GM and Neurodegenerative Diseases

The relationship between GM and neurodegenerative disease has recently gained a lot of attention in the medical community. An increasing number of studies suggested that GM can modulate nervous, endocrine, and immune communication through the gut-brain axis which takes part in the occurrence and development of central nervous system diseases, especially in Parkinson disease (PD) and Alzheimer disease (AD) (Collins et al., 2012).

### Parkinson Disease

PD is a common neurodegenerative disease of the central nervous system, which is characterized pathologically by the degeneration and loss of the dopaminergic neurons in the substantia nigra, along with the abnormal aggregation of α-synuclein in Lewy bodies (Beyer et al., 2009). Increasing evidence indicates a significant difference in GM composition between PD and healthy groups, albeit with an incongruent picture across different studies (Li C. et al., 2019; Elfil et al., 2020). Lots of studies showed that the abundance of *Bifidobacterium*, *Pasteurella*, and *Enterococcus* in intestinal microorganisms of PD patients increased significantly (Hill-Burns et al., 2017; Hopfner et al., 2017; Peng et al., 2018), while the abundance of *Brautella*, *Prevotella*, and *Faecococcus* decreased (Scheperjans et al., 2015; Bedarf et al., 2017; Heintz-Buschart et al., 2018).

Despite the findings of an increased *Bifidobacterium* in PD, a study showed that a decrease in *Bifidobacterium* later in disease may be able to predict whether PD stage is going to deteriorate or not. This implicates that the increase in *Bifidobacterium* in PD may represent a mechanism of beneficial effect against neurodegenerative aggravation. Thus, it can be inferred that a probiotic intervention with *Bifidobacterium* could prevent the progression of PD to severer stages (Gerhardt and Mohajeri, 2018).

However, the abundance of *Lactobacillus* in PD patients varied widely among different studies. The results are not altogether surprising, as the GM is affected by many factors such as different lifestyles, diet, age, geography, body mass index, and race (Li C. et al., 2019). Different countries have different diets which lead to a significantly different composition of *Lactobacillus* (Scheperjans et al., 2015). Even the changes of *Lactobacillus* are not consistent in different regions of the same country, such as in Southeast (Qian et al., 2018) and Southwest (Lin et al., 2018) China are different, which may be due to different dietary habits in different regions of China. Besides, the increase of *Lactobacillus* also could be caused by the frequent constipation of PD patients, since *Lactobacillus* could increase in constipation-type Irritable Bowel Syndrome and decreased in diarrhoea-type Irritable Bowel Syndrome (Gerhardt and Mohajeri, 2018).

The gut-brain axis, the two-way communication between intestinal flora and central nervous system, is involved in the regulation of brain function, neural development and aging (Fan and Pedersen, 2021). There are three main interaction pathways between gut microbiota and brain axis. The first is chemical signals. Bacteria can affect the nervous system directly or indirectly through SCFAs and other metabolites, acting on the neuroendocrine system, regulating the concentration of GABA and 5-HT and other neurotransmitters. The second is the neural pathways. Flora and their metabolites can act on the vagus and intestinal nervous system, affecting the brain and behavior. The last one is the immune system. Microglia and systemic cytokines (inflammatory level) have key mediating effects. These pathways may be involved in the pathogenesis of neurodegenerative diseases and other neuro related diseases (Morais et al., 2020).

Many experimental studies have confirmed gut-brain axis in PD. Alterations in GM in PD patients can lead to the accumulation of α-synuclein and excessive activation of microglia in brain neurons (Sampson et al., 2016). In an experiment that used mice overexpressing α-synuclein, it was shown that more α-synuclein aggregations were present in the brains of mice with non-eradicated microbial as compared to germ-free mice that had no gut bacteria. In the same study, transplantation of the fecal microbiota from PD patients to germ-free mice aggravated α-synuclein-induced motor symptoms to a greater degree than a fecal transplant from healthy people (Sampson et al., 2016). Recent studies show that the decline in short-chain fatty acids and ghrelin mediated by GM alteration in PD patients might be a critical factor in the pathophysiology of PD (Elfil et al., 2020).

A number of studies indicate a significant difference in GM composition between PD and healthy groups, albeit with an incongruent picture across different studies. Bacteria can affect the nervous system in PD directly or indirectly through chemical signals, neural pathways or the immune system. Therefore, these previous studies suggest that GM may play an important role in the intestinal lesions in the pathogenesis of PD.

### Alzheimer Disease

AD is the most prevalent neurodegenerative disease of the central nervous system and is characterized by β-amyloid protein deposition and hyperphosphorylation of tau in β-amyloid plaques or neurofibrillary tangles, which trigger the onset of the inflammatory response, leading to neuronal apoptosis or necrosis eventually irreversible damage to the brain (Maria et al., 2010). The role of intestinal microflora in the pathogenesis of AD is similar to that in PD, both of which begin with the imbalance of gut-brain axis (Abbott, 2020). A growing number of studies have shown the correlation between GM and AD (Kobayashi et al., 2017; Kim M. et al., 2020). Clinical studies indicated that intestinal flora is indeed involved in the early pathogenesis of human AD (Li B. et al., 2019). It was found that in the mild cognitive impairment stage, the changes of intestinal flora were similar to those in the onset stage of AD, that is, the diversity of fecal flora was significantly lower than that in healthy people. This indicates that dysbacteriosis has the potential for early diagnosis of AD. An increasing number of studies indicated that AD is tightly associated with the inflammation-driven by GM. The dysbiosis of GM in AD patients might decrease the abundance of anti-inflammatory bacteria, and increase the abundance of specific pro-inflammatory bacteria including *Escherichia* and *Shigella* (Minter et al., 2017). A large number of metabolites produced by pro-inflammatory gut bacteria taxa can drive the invasion of peripheral immune cells and elevate the level of inflammation in plasma and central nervous system (Kobayashi et al., 2017; Mancuso and Santangelo, 2017). Compared with healthy wild-type mice, AD mice show changes in intestinal flora composition, loss of epithelial barrier integrity, chronic intestinal and systemic inflammation (Kim M. et al., 2020).

In recent years, the theory that microbial infection promotes the pathogenesis of AD has been proposed. Abbott et al. have found that herpes simplex virus type 1 infection can be detected in the brains of AD patients. Through further study, they found that the existence of amyloid beta plaques is a kind of protective protein against virus infection, which can directly surround and engulf the virus invading the brain. These “protective” proteins remain in the brain and can not be cleaned up in time, and eventually cause great damage to the nerve (Abbott, 2020). This study suggests that microbial infections may be responsible for AD.

In addition to microbial infection, amyloid beta protein aggregation in the brain to form plaques and cause nerve damage is one of the main pathogenic factors of AD. An increasing number of researches reported that GM affects the severity of β-amyloid protein pathology and cognitive impairment in AD models. For example, rearing APPPS1 mice in germ-free conditions resulted not only in a significant reduction of neuronal β-amyloid protein accumulation but also a large decrease in Iba-1-positive microglia leading to an overall decrease in neuroinflammation compared to conventionally-raised (Harach et al., 2017). Similarly, transplantation of the fecal microbiota from healthy mice into AD mouse models ameliorated the formation of β-amyloid protein plaques and neurofibrillary tangles, glial reactivity and cognitive impairment (Kim M. et al., 2020). The possible pathogenesis includes the imbalance of intestinal flora with aging, which can activate the CCAAT/enhancer binding protein β/asparagine endopeptidase pathway in the gut and brain, thus promoting the occurrence and development of AD. Further studies showed that the prebiotic R13 can inhibit this pathway by enriching specific probiotics and suppresses amyloid aggregates in the gut (Chen et al., 2020). Furthermore, a recent study reported that the alteration of GM composition leads to the circulating accumulation of phenylalanine and isoleucine, which further stimulates the differentiation and proliferation of pro-inflammatory T helper 1 cells. In the same study, experimental verification has further confirmed that GV-971 could inhibit peripheral phenylalanine and isolucine accumulation, control neuroinflammation, and improve cognitive impairment (Wang et al., 2019).

The role of intestinal microflora in the pathogenesis of AD patients begins with the imbalance of gut-brain axis. An increasing number of studies indicated that AD is tightly associated with the inflammation driven by GM. From the above, it is clear that GM dysbiosis can promote neuroinflammation in AD progression and remodel the dynamic balance of GM, which may be a novel strategy for AD therapy. Regulation of intestinal flora may have a certain application prospect in the treatment of nervous system diseases.

## GM and Cardiovascular Diseases

Cardiovascular diseases (CVD), such as hypertension, atherosclerosis and heart failure, are a major cause of death worldwide (Murray et al., 2015). A growing number of studies have exposed that GM and its metabolic products interact with the host in many different ways to influence the development and occurrence of CVD. The role of Trimetlylamine oxide (TMAO), Bile acids, and SCFAs in CVD has been supported by a large number of studies, which are metabolic products of the gut microbiome (Sanchez-Rodriguez et al., 2020).

### Hypertension

Hypertension is one of the most common risk factors of CVD (Collaboration, 2017). Hypertension is associated with altered gut function, altered gut bacterial populations and changes in gut–nervous system connectivity. In hypertensive patients, microbial richness, diversity, and evenness were significantly decreased (Yang et al., 2015; Yan et al., 2017) while the ratio of *Firmicutes*/*Bacteroidetes* ratio is remarkably increased (Li J. et al., 2017). The change of intestinal flora plays a key role in the regulation of blood pressure, and the change in the production of gut microbial metabolites may be a key mechanism. Based on the results of a meta-analysis of 18 observational studies, it was found that circulating (serum or plasma) TMAO concentration was significantly dose-dependent associated with hypertension risk and was associated with cardiovascular metabolic risk indicators such as HDL cholester (Abbasalizad Farhangi and Vajdi, 2020).

The effect of GM goes far beyond regulating the gut itself. GM metabolites can be absorbed into the blood and transported through the blood-brain barrier to regulate brain function. The imbalance of SCFA caused by GM dysbiosis can stimulate intestinal chromaffin cells to produce 5-hydroxytryptamine (5-HT), which can act on the 5-HT3 receptor of intestinal vagus nerve and inhibit the afferent activity of vagus nerve from intestine to brain. 5-HT released into blood can lead to vasoconstriction. Then 5-HT and SCFA can affect blood pressure through blood circulation and blood brain barrier damage caused by hypertension (Zubcevic et al., 2019). In addition, Hydrogen sulfide (H2S) produced by GM has an important impact on human health. H2S can regulate a variety of physiological processes, including vasorelaxation, angiogenesis, and hypotension (Nagpure and Bian, 2016). H2S deficiency occurs before the occurrence of hypertension. Exogenous H2S donor can protect spontaneously hypertensive rat from hypertension, which indicates that H2S deficiency plays an important role in the regulation of blood pressure (Donertas Ayaz and Zubcevic, 2020). H2S may improve the ability of hypertension through a variety of mechanisms, including inhibition of oxidative stress or inflammation and ion channel mediated relaxation of vascular smooth muscle (Meng et al., 2015; Zeng and Tan, 2020).

Recently, growing evidence has suggested that intestinal dysbacteriosis is involved in the regulation of inflammatory processes, vascular permeability, and blood pressure (Li X. S. et al., 2017; Robles-Vera et al., 2017). GM facilitates angiotensin II-induced hypertension and vascular dysfunction through vascular immune cell infiltration and inflammation (Karbach et al., 2016). Animal experiments concluded that the introduction of hypertensive patients donor stool into the bowel of mice resulted in elevated values of blood pressure, suggesting a causal role of the GM in the development of hypertension (Durgan, 2017; Shikata et al., 2019; Toral et al., 2019). Another study reported that antibiotic treatment in rats can rebalance the dysbiotic hypertension GM by reducing the *Firmicutes/Bacteroidetes* ratio, which then attenuates high blood pressure (Yang et al., 2015). Furthermore, the ablation of the entire GM *via* an antibiotic cocktail remarkably reduced the incidence of hypertension-related aneurysms suggesting that GM contributes to the pathophysiology of aneurysms by regulating inflammatory and immune response (Shikata et al., 2019). A randomized controlled trial found that dietary intervention rich in polyphenols can significantly improve the intestinal permeability of the elderly, increase the number of cellulolytic and butyrate producing bacteria in the intestinal flora, and reduce blood pressure (Del Bo et al., 2020).

The richness, diversity, and evenness of intestinal microbes were significantly reduced in patients with hypertension. The effect of GM on hypertension is not only through the regulation of intestinal tract itself but also through a change in the production of its metabolites. These findings highlight a strong linkage between hypertension and GM, suggesting that correcting the balance of GM could be a promising therapeutic strategy for hypertension.

### Atherosclerosis

Atherosclerosis, which accounts for approximately 50% of CVD, is the principal cause of coronary heart disease, cerebral infarction and peripheral vascular disease (Sanchez-Rodriguez et al., 2020). Recent research established a possible link between GM and CVD by evidence of bacterial translocation from the gut to the heart, and both live oral bacteria and bacterial DNA were detected in atherosclerotic plaques, indicating that GM is involved in the development and progression of atherosclerosis (Kozarov et al., 2005; Mitra et al., 2015; Jonsson and Bäckhed, 2017). A metagenome-wide association study found that GM composition varies significantly among atherosclerosis patients and healthy controls. The abundance of *Enterobacteriaceae* and *Enterobacter aerogenes* is much higher in atherosclerosis patients than in control samples, which inhibited the growth of beneficial bacteria (Li et al., 2016; Jie et al., 2017). Meanwhile, several animal models and human studies have demonstrated that intestinal dysbacteriosis in atherosclerosis could increase intestinal permeability, and thereby increase lipopolysaccharide absorbed into circulation (Zhang et al., 2020). Thus, a distant infection or a direct infection of vessel wall cells could affect the development of atherosclerotic plaques (Jonsson and Bäckhed, 2017).

In addition, the implications of gut microbiota and its metabolites can also affect atherosclerosis development. The human GM can produce a wide variety of enzymes that ferment dietary fibers into SCFAs such as acetate, propionate, and butyrate. SCFAs as signal molecules activate G-protein-coupled receptors mainly include GPR41 and Olfr78, which can promote the release of peptide YY (PYY) and glucagon-like peptide 1 (GLP-1), thus reduce blood pressure and inhibit the occurrence of atherosclerosis (Zeng and Tan, 2020). Bile acids, as the end product of cholesterol catabolism, have an anti-atherosclerotic effect. BAs are one of the major classes of metabolites modified by GM, and they can activate the Farnesoid X receptor (FXR) then reduce the expression of inflammatory factors on monocytes, macrophages and dendritic cells, and reduce the level of inflammation, thus inhibiting the occurrence of atherosclerosis (Wang et al., 2011; De Palma et al., 2015; Bharwani et al., 2017). TMAO is another metabolite of GM with a major role in the formation of atherosclerosis. Epidemiologic studies have demonstrated that TMAO has become a new indicator to identify the risk of cardiovascular events (Skye et al., 2018). A positive correlation between high-level TMAO and the incidence of atherosclerosis has been indicated (Nie et al., 2018). GM metabolized choline, betaine, and carnitine to produce trimethylamine, which is further delivered to the liver where flavin monooxygenase converts it to TMAO (Zhu et al., 2020). High levels of TMAO promote the migration of macrophages and accelerate the transformation of macrophages into foam cells, increasing the expression of pro-inflammatory cytokines, thereby promoting the formation of atherosclerosis (Wang et al., 2011; Koeth et al., 2013). TMAO also impairs vascular reactivity and causes oxidative stress, leading to endothelial dysfunction and result in atherosclerosis (Haghikia et al., 2018; Chou et al., 2019). Besides, TMAO can also activate platelets to promote thrombosis and atherosclerotic plaque rupture (Zhu et al., 2016). Recently, Nemet et al. identified phenylalanine as phenylacetylglutamine (pagln) by non-targeted metabonomics. Pagln is a gut microbiota-derived metabolite that can enhance platelet activation-related phenotypes and thrombosis potential. Pagln acts on G protein coupled receptors, including α2a, α2B, and β2-adrenergic receptors, to induce downstream cellular responses. Through further study, they found that pagin can increase the potential of thrombosis through G protein coupled receptor (Nemet et al., 2020).

GM composition varies significantly among atherosclerosis patients and healthy people. Further research revealed that GM was involved in the development and progression of atherosclerosis, as bacterial DNA was detected in atherosclerotic plaques. Intestinal microorganisms and their metabolites can influence the development of atherosclerosis. Microorganisms metabolized some alkaloids into harmful substances, which further activate pro-inflammatory cytokines, thereby promoting the formation of atherosclerosis. Overall, these studies illustrate that GM and its metabolic products can regulate the occurrence and development of atherosclerosis through multiple pathways. Regulating GM can be one of the therapeutic strategies for cardiovascular health.

## GM and Metabolic Diseases

Although the incidence of metabolic diseases is related to genetic and environmental factors, the incidence of metabolic diseases in people with the same genetic background and energy intake is correlated with the presence of intestinal flora. In recent years, increasing studies have suggested that GM dysbiosis is closely associated with many metabolic disorders including obesity, diabetes, and non-alcoholic fatty liver disease (NAFLD).

### Obesity

Obesity and its metabolic complications are major public health problems, more than 1.9 billion adults are overweight and over 650 million adults are obese (World Health Organization, 2020). More and more studies have demonstrated that GM plays an important role in the development of obesity (De La Cuesta-Zuluaga et al., 2018). The GM dysbiosis is prevalent in obesity, which is manifested as a reduction in gut microbiome diversity and richness in obese. The abundance of *Akkermansia muciniphila, Faecalibacterium prausnitzii*, and *Bacteroides* decreased, while the abundance of *Phylum Firmicutes* increased significantly (Vallianou et al., 2019). In 2006, Turnbaugh et al. found that intestinal microecological is different between lean and obese people, and further found that germ-free mice transplanted with GM from obesity mice gained higher fat and more weight than lean mice, suggesting that GM dysbiosis may contribute to obesity (Turnbaugh et al., 2006). A metagenome-wide association study in a cohort of lean and obese in Chinese adolescents found that the abundance of *Bacteroides thetaiotaomicron* significantly decreased in obese people, and negatively correlated with serum glutamate concentration (Liu et al., 2017). Animal experiments have confirmed that gavage with *B. thetaiotaomicron* reduced serum branched chain amino acid concentrations and alleviated diet-induced weight gain and obesity in mice (Zeng et al., 2020). Moreover, bariatric surgery induced changes to the gut microbiome that could last for a decade, and induced a reduction of *B. thetaiotaomicron* (Liu et al., 2017), suggesting that intervention in obesity by targeting the GM might be possible.

In addition, GM has the ability to ferment indigestible carbohydrates to the important metabolites such as SCFAs and succinate. Recent studies indicated that these metabolites play a significant role in obesity and its comorbidities. SCFAs regulate energy balance and prevent obesity by suppressing appetite and increasing energy expenditure (Canfora et al., 2019). GLP-1 and PYY by enteroendocrine L-cells have a link to increased satiety and reduced food intake (Tischmann et al., 2019). SCFAs can regulate GLP-1 and PYY by regulating immune cell function through receptors GPR41 and GPR43 (Larraufie et al., 2018). Additionally, SCFAs can also induce the up-regulation of heat-generating proteins (PPARγ, PGC1α, UCP1) and lipid oxidation-related proteins (CPT-I, UCP2), thereby increasing energy consumption and lipid oxidation to prevent obesity (Sittipo et al., 2019). Succinat is produced by gut bacteria from the phylum *Bacteroidetes* could enhance intestinal gluconeogenesis by binding to the GPR91 receptor and thereafter activate gut-brain glucose signaling which affects adiposity and body weight at the end. In a study of experimental animals, De Vadder et al. found that succinate is a critical molecule produced intrinsically by *Prevotella* in the regulation of glucose metabolism (De Vadder et al., 2016). After supplementation with oligofructose, the cecal succinate concentration of mice increased, leading to the activation of gluconeogenesis, thereby preventing the obesity and glucose tolerance phenotype of mice.

The imbalance of intestinal flora, such as decreased diversity and richness of intestinal flora, is common in obese people. Some of the products from GM fermented indigestible carbohydrates prevent obesity by inhibiting appetite and increasing energy consumption, while others prevent obesity by increasing energy consumption and lipid oxidation. The above studies have clarified the role and mechanism of GM and its metabolites in the occurrence and development of obesity, which are of great significance for obesity prevention and treatment.

### Diabetes

Diabetes is a systemic metabolic disease characterized by high blood glucose, including type 1 diabetes mellitus (T1DM), type 2 diabetes mellitus (T2DM), gestational diabetes mellitus (GDM). There is accumulating evidence that GM is a risk factor in the occurrence and development of diabetes.

T1DM is an insulin-dependent type of diabetes (Pan et al., 2018). Although the exact causes of T1DM remain unknown, it is clear that both genetic and environmental factors contribute to the development of T1DM (Bakay et al., 2019). Between them, GM has become one of the key environmental factors. Several studies have found that there are significant differences in the gut microbial composition between T1DM patients and healthy people (Zhou et al., 2020a). The diversity of GM in T1DM patients is reduced, which is manifested by the decreased amount of *Clostridium* and *Prevotella* (Zhou et al., 2020b). Non-obese diabetic mice can produce spontaneous T1DM. Transplanting the fecal flora of non-obese diabetic mice into anti-diabetic mice can induce increased pancreatitis inflammation in anti-diabetic mice (Brown et al., 2016). This indicates that GM affects the pathological process of T1DM to a certain extent.

T2DM is non-insulin-dependent diabetes, which is characterized by a decline in insulin secretion and insulin resistance (Chatterjee et al., 2017). A Swedish cohort study found that the gut microbial diversity of T2DM patients was significantly reduced (Wu H. et al., 2020). Compared with healthy individuals, the abundance of *Bifidobacteria* and *Akkermansia* in the gut of T2DM patients is decreased, while the abundance of *Dallella* is increased (Li et al., 2020). Epidemiological studies have found that patients with colectomy have an increased risk of T2DM compared with non-colectomy patients (Jensen et al., 2018), which indirectly proves that GM and hormone secretion in the distal intestine may participate in the regulation of glucose metabolism. A large prospective cohort study analyzed the diversity and composition of GM in different subgroups, found that the increase in the level of γ-linolenic acid was positively correlated with the risk of T2DM, while the baseline γ-linolenic acid level was significantly negatively correlated with the gut microbial richness and diversity during follow-up (Miao et al., 2020). The study suggests that GM mediates the association between γ-linolenic acid and T2DM. The above studies show that there is a correlation between T2DM and changes in GM.

GDM is a common complication during pregnancy, which is associated with an increase of adverse pregnancy outcomes (Hasain et al., 2020). Several studies have found that the intestinal microbial disorders of GDM patients are manifested by an increase in *Rumenococcus*, *Desulfovibrio*, *Enterobacter*, *Bacteroides*, and *Prevotella*, while a decrease in *Bifidobacterium* and *Fischeri* (Crusell et al., 2018). Interestingly, 8 months after pregnancy, differences in the GM signatures are still detectable (Crusell et al., 2018). GM dysbiosis in GDM patients is associated with inflammation, obesity, and impaired glucose tolerance. A metagenomics study found that the distribution of metagenomic linkage groups in GDM patients is different from controls, and functional analysis shows that the microbiome of GDM patients has a higher abundance of membrane transport, energy metabolism pathways, lipopolysaccharide (LPS) and phosphotransferase system (PTS), while the PTS pathway and lipopolysaccharide signaling pathway is correlated with the level of glucose tolerance (Kuang et al., 2017). In addition, the latest systematic review showed that supplementing with probiotics could regulate GM to prevent GDM (Homayouni et al., 2020).

Many studies have found that the metabolites of GM are also involved in the occurrence and development of diabetes. Metabolites such as lipopolysaccharide, flagellin, lipoteichoic acid and peptidoglycan can destroy the tight junctions between intestinal epithelial cells, and induce inflammation through TRL2 and TRL4 signals (Ebrahimzadeh Leylabadlo et al., 2020). High levels of branched chain amino acids and TMAO can lead to increased insulin resistance and inflammation (Ebrahimzadeh Leylabadlo et al., 2020). Elevated levels of SCFAs can promote the secretion of GLP-1 and PYY to prevent intestinal transit and insulin resistance, and stimulate the secretion of glucagon-like peptide-2, which lead to decreased intestinal barrier function, endotoxemia, and inflammation (Aoki et al., 2017). BAs bind to the TGR5 signal receptor to activate intestinal cells to secrete GPL-1, which ultimately leads to increased insulin sensitivity and glucose uptake (Jahansouz et al., 2016). Recently, a team of Chinese scholars made a breakthrough progress in the mechanism of small molecule metabolites regulating blood glucose. It was found that hycholic acid (HCA) can effectively regulate the blood glucose balance and has the effect of treating type 2 diabetes. Through activating TGR5 and inhibiting FXR signal, HCA promotes the production and secretion of GLP-1 by intestinal endocrine cells, thus controlling the level of blood glucose. Analysis of clinical samples also showed that serum HCA was negatively correlated with diabetes and blood glucose levels. However, as the bile acids with less attention, HCA is expected to become potential therapeutic drugs for diabetes, and can also be combined with other hypoglycemic drugs to improve clinical efficacy (Zheng et al., 2020). At the same time, because the metabolism of cholic acid is regulated by intestinal flora, how to improve the level of cholic acid in diabetic patients through intestinal flora will also become the focus of future research and drug development.

On the other hand, GM can also autonomously regulate blood glucose through enteric nerves (Muller et al., 2020). The distribution of GM in the digestive tract is not uniform. Intrinsic enteric-associated neurons can monitor diet and commensal microbe signals to regulate intestinal motility and secretion functions (Veiga-Fernandes and Mucida, 2016). Studies have proved that intrinsic enteric-associated neurons in different intestinal segments of mice are flora-dependent, and intestinal microbes regulate CART neurons to regulate blood glucose levels independently from the central nervous system (Muller et al., 2020).

T1DM diabetes is influenced by GM as well as genetic factors. The gut microbial composition is significant differences between T1DM patients and healthy people. The diversity of GM in T1DM patients is reduced. Transplanting the fecal flora from healthy people may be a new method to treat T1DM diabetes in the future. GM can also autonomously regulate blood glucose through enteric nerves to monitor diet and commensal microbe signals to regulate intestinal motility and secretion functions. The discovery of this peripheral neural circuit provides a new perspective for understanding the role of GM in tissue physiology, metabolism, immunity, and nervous system functions. In the future, it may be possible to treat diabetes by regulating gut microbial metabolites or microorganism species.

### Non-Alcoholic Fatty Liver Disease

NAFLD is an important chronic disease, which can develop into liver cirrhosis and liver cancer without intervention and control. The prevalence of NAFLD is rising as the obesity pandemic expands. NAFLD is a multivariate disease involving both environmental and genetic factors. Although the molecular mechanism underlying NAFLD occurrence and progression remains imperfectly understood, evidence from studies shows that GM is closely related to NAFLD.

The integrity of the gut barrier is critical to protect the liver from the invasion of intestinal microflora. A meta-analysis found that intestinal permeability in patients with NAFLD was higher than that in healthy controls and was associated with the degree of hepatic steatosis (De Munck et al., 2020). Besides, the composition of gut microbes is different between NAFLD patients and healthy people. The abundance of *Lactobacillus*, *Dorea*, *Streptococcus* in the intestine of NAFLD patients increased, while the presence of *Rumenococcus*, *Prevotella*, and *Flavobacterium* decreased (Raman et al., 2013; Jiang et al., 2015). Fecal transplantation showed that mice transplanted with stool from NAFLD patients had higher levels of triglyceride content in mouse liver and thereby triggered liver steatosis (Hoyles et al., 2018), which indicates that changes in gut microbes play an important role in NAFLD (Wang et al., 2018).

GM metabolites derived from saccharolytic and proteolytic fermentation might affect the gut–liver axis *via* multiple mechanisms and hence lead to the pathogenesis of NAFLD (Canfora et al., 2019). SCFAs have beneficial effects on liver metabolism and function. Butyrate especially improves the intestinal barrier function and prevents toxic compounds to the liver by up-regulating the expression of tight junction proteins and mucus (Kelly et al., 2015). The potential underlying mechanism is related to increased hepatic lipid oxidation through an AMPK–acetyl-CoA carboxylase pathway, reducing tumor necrosis factor expression, increasing glycogen storage and reducing liver fatty acid synthase activity (Mollica et al., 2017).

The GM can also inhibit hepatic lipid synthesis by regulating BAs metabolism to alter FXR signaling pathways (Iruzubieta et al., 2020). Studies have found that glycine may be directly related to the occurrence of NAFLD. Glycine significantly alleviates NAFLD in experimental mice by regulating fat oxidation, blood metabolite levels, gut microbiota composition, and glutathione synthesis (Rom et al., 2020). Furthermore, fibroblast growth factor-15/19 is a bile acid-induced gut hormone in the late fed phase that acts to decrease hepatic lipogenesis. Fibroblast growth factor-15/19 suppresses hepatic lipogenesis through an epigenetic mechanism by activating its downstream nuclear receptor small heterodimer partner, which in turn recruits the DNA methyltransferase DNA methyltransferase-3a to lipogenic gene loci such as fatty acid synthase (Kim Y. C. et al., 2020). This study may shed new light on protection against metabolic diseases.

In addition, endogenous ethanol (Chu et al., 2019) produced by intestinal microbial can also be involved in NAFLD progression through direct toxic effects on liver cells (Xu et al., 2011). Endogenous ethanol can lead to increased portal endotoxemia through impairments in gut barrier function and up-regulated signaling pathways in peripheral cells through the nuclear factor-κB pathway (You and Arteel, 2019). The above studies showed that the interaction between gut microbes and the liver plays a crucial role in the occurrence and development of NAFLD. In addition to genetic factors, people with NAFLD have a different gut microbiome composition than healthy people. Some GM metabolites might affect the gut-liver axis *via* multiple mechanisms and hence lead to the pathogenesis of NAFLD.

## GM and Gastrointestinal Diseases

In the long evolutionary process, intestinal flora continuously adapts to individual adaptation and natural selection, and interacts and restricts with the host to regulate intestinal homeostasis. Intestinal microorganisms absorb nutrients from the host, degrade food residues that cannot be digested by the host, and participate in the metabolism of nutrients in the gut (Zhang et al., 2018). Accumulating evidence suggests that GM dysbiosis can cause digestive system diseases, including inflammatory bowel diseases (IBD) and colorectal cancer (CRC).

### Inflammatory Bowel Disease

IBD consisting of ulcerative colitis and Crohn’s are resultant of dysregulation of the immune system leading to intestinal inflammation and microbial dysbiosis (Caruso et al., 2020). Various evidence indicated that GM contributes to driving intestinal inflammation. A loss of gut microbial diversity as one of the symbols of dysbiosis is commonly found in IBD patients. Compared with healthy individuals, the IBD GM displays a marked reduction in gut microbial diversity, which is manifested as a significant decrease in *Firmicutes* while a significant increase in *Enterobacter* and *Proteobacteria* (Caruso et al., 2020). In addition to bacteria, fungi have also changed in the intestines IBD patients (Da Silva et al., 2018). The diversity of fungi in the colon, ileum and feces of patients with Crohn’s disease increased remarkably, manifested by an increase in *Candida albicans*, *Aspergillus albicans*, and *Cryptococcus neoformans* (Li et al., 2014). Experimental animal studies further supported the idea that the participation of GM in the pathophysiology of IBD. After transferring the gut microbes of IBD mice to germ-free mice, germ-free mice showed clinical symptoms of inflammatory bowel disease (Schaubeck et al., 2016). Similarly, C Eftychi et al. found that a combination of specific symbiotic flora can trigger colitis in mice with impaired intestinal barrier function (Eftychi et al., 2019). The above studies show that inflammatory enteritis is related to certain intestinal microbes, but the specific microbial population needs further study.

In addition, metabolites of gut microbes also play a role in the pathogenesis of IBD. SCFAs mediate multiple effects on mucosal immunity by promoting B cell development, maintenance of mucosal integrity *via* inflammasome activation and IL-18 production (Macia et al., 2015). BAs play an immunomodulatory role by direct stimulation of FXR, which exerts anti-inflammatory effects and protects in chemically induced colitis (Lavelle and Sokol, 2020). Tryptophan is utilised by GM to produce indoles, which can regulate mucosal immunity by activating polycyclic aromatic hydrocarbon receptors (Yano et al., 2015). Furthermore, gut microbes can also regulate host cell functions through epigenetics. The latest study indicated that inositol triphosphate, a metabolite of GM, can antagonize the inhibitory effect of butyric acid on histone deacetylase 3 and induce the activation of protein deacetylase 3, thereby promoting intestinal epithelial cell proliferation and intestinal injury repair and improve inflammatory bowel disease (Wu S. E. et al., 2020).

The above studies have shown that GM is a precipitating factor in the occurrence and development of IBD, but its specific mechanism remains to be verified. Therefore, it is necessary to further reveal the relationship between GM and IBD, so as to provide a new direction for the treatment of IBD.

### Colorectal Cancer

Colorectal cancer (CRC) is one of the most common malignancies in the digestive system. Studies have shown that GM dysbiosis is related to CRC. Intestinal microbial Dysbiosis in CRC patients is characterized by a decrease in the species of intestinal probiotics (such as *Bifidobacteria*, *Lactobacillus*, and *Bacteroides*) and an increase in the number of pathogenic bacteria (such as *Escherichia coli*, *Bacteroides fragilis*, and *Fusobacterium nucleatum*). The pathogenic bacteria secrete toxic chemicals that damage intestinal epithelial cells and cause a chronic inflammatory response, which contributes to the development of CRC (Si et al., 2020).

*In vitro* and *in vivo* experiments have further investigated the mechanism of GM promoting tumor occurrence and development. Tsoi H et al. found that *Peptostreptococcus anaerobius (Pa) *is significantly enriched in feces samples and colon tumor tissues of patients with CRC, which can promote the abnormal proliferation of colon cells (Tsoi et al., 2017). Cell and animal experiments further explained the mechanism of *Pa* promoting CRC. They found that *Pa* can bind to the integrinα2/β1 on the surface of CRC cells through its surface protein PCWBR2, and activate the downstream PI3K-Akt-NF-κB signaling pathway, thereby driving the occurrence of CRC (Wong et al., 2017; Long et al., 2019). Research by Garrett WS et al. found that *Fusobacterium nucleatum* can bind cadherin and inhibitory immune receptor TIGIT on the surface of CRC cells through adhesin FadA and Fap2, respectively, to activate oncogenic Wnt/beta-catenin signaling pathway and inhibit the function of tumor infiltrating lymphocytes and natural killer cells, thereby promoting the development of CRC (Garrett, 2019). In addition, studies have found that GM can also change certain biological functions of host genes. In the mouse model of intestinal cancer, p53 mutants have different effects in different parts of the intestine. The p53 mutant has the expected carcinogenic effect in the distal intestine, but it can significantly inhibit the occurrence and development of tumors in the proximal intestine. Further research exposed that Gallic acid, a metabolite of intestinal microbes, can transform mutant p53 from a tumor-inhibiting effect to a carcinogenic effect. It suggests that gut microbes might interact with the existence of cancer mutant genes (Kadosh et al., 2020). A newly published study showed that gut microbes can induce symbiotic specific memory T cells that cross-react with tumor antigens, and specific *Enterococci* phages use this molecular mechanism to enhance the efficacy of anti-tumor immunotherapy (Fluckiger et al., 2020).

The above studies have highlighted that the toxic metabolites produced by GM can directly participate in the occurrence of cancer, or indirectly participate in cancer through inflammation or immunosuppression. Furthermore, intestinal microbial disorders also play a crucial role in the occurrence of CRC.

## Discussion

GM is closely related to human health and diseases, which bring great opportunities for the diagnosis, treatment, and prevention of diseases. This paper describes the research progress of the pathogenesis and mechanism of GM in multi-system diseases. For the past few years, metagenomic research has become a popular research method to study the correlation between gut microbes and diseases. Obtaining high-quality reference genome sets can significantly improve the resolution and accuracy of metagenomic research, and provide a data basis for the correlation analysis between the human gut microbiome and phenotype. The latest research has established a gene set of 200,000 genomes and 171 million protein sequences representing the human gut microbiome. Among 4,644 species, 71% of the strains lack cultivation, and high strain variability between continents, which shows that most microorganisms still need experimental research (Almeida et al., 2020). At the same time, there are still many unsolved mysteries in the research of the relationship between gut microbes and diseases. Firstly, there is no consensus on what is a “good” gut microbiome. How to define intestinal microbial disorders clinically, and develop reliable microbiota screening methods for accurate diagnosis, to recognize people with intestinal microbial disorders? Secondly, it is still unclear whether the intestinal microbial disorder is the cause or the result of the disease, or whether it is a specific strain of bacteria, or the overall intestinal microbe as the cause of the disease. The mechanism of GM involved in the occurrence and development of diseases is not yet clear. Furthermore, can intestinal microbes that are beneficial to disease treatment prevent disease and promote overall immune health? Finally, the interactions between gut microbes, host immune systems, and diseases are not only complex but also highly dynamic, this may mean that different gut microbiota treatments are needed to be adopted throughout the course of disease treatment. At present, there are few effective methods for the treatment of clinical diseases by regulating intestinal flora. Therefore, it is very necessary to combine advanced techniques such as metagenomics, transcriptomics, proteomics, and metabolomics to conduct prospective studies with large samples. In the context of precision medicine, it is possible to use personalized, genetically modified microbiota for the prevention and treatment of certain diseases in the future. Targeting the composition and metabolic function of intestinal flora may be a new option for the prevention and treatment of diseases.

In conclusion, there are great opportunities for studies at all levels, from basic and translational research to clinical and epidemiological analysis, which can advance the understanding of this complex intestinal ecosystem. This requires reasonable experimental design and long-term dynamic tracking of changes in intestinal microbes and disease development, combined with multi-omics analysis and more comprehensive high-throughput sequencing. Go deeper into the single strain level of research, so as to find disease-related conditional pathogenic bacteria. It may provide new ideas for disease treatment and give full play to the potential of precision medicine.

## Author Contributions

YC contributed to conception, design, and drafting the manuscript. JZ and LW contributed to conception, design, and critically revised the manuscript. All authors contributed to the article and approved the submitted version.

## Funding

This work was supported by the National Natural Science Foundation of China (No. 31701271) and the Natural Science Foundation of Zhejiang Province (No. LY21C110001). The funders had no role in study design, data collection and analysis, decision to publish, or preparation of the manuscript.

## Conflict of Interest

The authors declare that the research was conducted in the absence of any commercial or financial relationships that could be construed as a potential conflict of interest.
