# Supplementary material for: Role and Mechanism of Gut Microbiota in Human Disease
Source: Front Cell Infect Microbiol. 2021 Mar 17;11:625913. doi: 10.3389/fcimb.2021.625913 (PMC8010197; doi:10.3389/fcimb.2021.625913)
Supplement: Supplementary file 1 [file Table_1.docx]

|  | **Disease** | **Up-regulated** | **Down-regulated** | **References** |
| --- | --- | --- | --- | --- |
| Neurodegenerative Diseases | Parkinson disease | *Bifidobacterium* | *Brautella* | Hill-Burns et al., (2017); Hopfner et al., (2017); Peng et al., (2018); Scheperjans et al., (2015); Bedarf et al., (2017); Heintz-Buschart et al., (2018) |
|  |  | *Pasteurella* | *Prevotella* |  |
|  |  | *Enterococcus* | *Faecococcus* |  |
|  | Alzheimer disease | *Escherichia* | *anti-inflammatory bacteria* | Minter et al., (2017) |
|  |  | *Shigella* |  |  |
| Cardiovascular Diseases | Hypertension | *Prevotella* |  | Yan et al.,(2017); Li B. et al.,(2017); Yang et al.,(2015) |
|  |  | *Klebsiella* |  |  |
|  |  | *Streptococcus* |  |  |
|  |  | *Firmicutes/ Bacteroidetes* |  |  |
|  | Atherosclerosis | *Enterobacteriaceae* |  | Jie et al., (2017); Li et al.,(2016) |
|  |  | *Enterobacter aerogenes* |  |  |
|  |  | *Collinsella* |  |  |
|  |  | *Anaeroglobus* |  |  |
| Metabolic Diseases | Obesity | *Mollicutes* | *Akkermansia* | Turnbaugh et al., (2009); Liu et al., (2017); Vallianou et al., (2019) |
|  |  | *Phylum Firmicutes* | *Faecalibacterium* |  |
|  |  |  | *Oscillibacter* |  |
|  |  |  | *Alistipes* |  |
|  |  |  | *Bacteroides thetaiotaomicron* |  |
|  | T1DM |  | *Clostridium* | Zhou et al., (2020b) |
|  |  |  | *Prevotella* |  |
|  | T2DM | *Dallella* | *Bifidobacteria* | Li et al., (2020) |
|  |  |  | *Akkermansia* |  |
|  | GDM | *Rumenococcus* | *Bifidobacterium* | Crusell et al., (2018) |
|  |  | *Desulfovibrio* | *Fischeri* |  |
|  |  | *Enterobacter* |  |  |
|  |  | *Bacteroides* |  |  |
|  |  | *Prevotella* |  |  |
|  | NAFLD | *Bacteroidetes* | *Firmicutes* | Da Silva et al., (2018); Raman et al., (2013); Jiang et al., (2015) |
|  |  | *Lactobacillus* | *Rumenococcus* |  |
|  |  | *Dorea* | *Prevotella* |  |
|  |  | *Streptococcus* | *Flavobacterium* |  |
| Gastrointestinal Diseases | IBD | *Enterobacter* | *Firmicutes* | Caruso et al., (2020); Li et al., (2014) |
|  |  | *Candida albicans* | *Bifidobacterium* |  |
|  |  | *Aspergillus albicans* | *Roseburia* |  |
|  |  | *Cryptococcus neoformans* | *Faecalibacterium prausnitzii* |  |
|  |  | *Proteobacteria* | *Saccharomyces cerevisiae* |  |
|  | CRC | *Escherichia coli* | *Bifidobacteria* | Si et al., (2020); Tsoi et al., (2017) |
|  |  | *Bacteroides fragilis* | *Lactobacillus* |  |
|  |  | *Fusobacterium nucleatum* | *Bacteroidetes* |  |
|  |  | *Peptostreptococcus anaerobius* |  |  |
